# Determination of key enzymes for threonine synthesis through in vitro metabolic pathway analysis

**DOI:** 10.1186/s12934-015-0275-8

**Published:** 2015-06-13

**Authors:** Yanfei Zhang, Qinglong Meng, Hongwu Ma, Yongfei Liu, Guoqiang Cao, Xiaoran Zhang, Ping Zheng, Jibin Sun, Dawei Zhang, Wenxia Jiang, Yanhe Ma

**Affiliations:** Key Laboratory of Systems Microbial Biotechnology, Tianjin Institute of Industrial Biotechnology, Chinese Academy of Sciences, Tianjin, 300308 People’s Republic of China; Key Laboratory of Systems Bioengineering, Ministry of Education, Department of Biochemical Engineering, School of Chemical Engineering and Technology, Tianjin University, Tianjin, 300072 People’s Republic of China

**Keywords:** Key enzyme, Metabolic control analysis, Threonine synthesis, Proteomics, Flux control coefficient

## Abstract

**Background:**

The overexpression of key enzymes in a metabolic pathway is a frequently used genetic engineering strategy for strain improvement. Metabolic control analysis has been proposed to quantitatively determine key enzymes. However, the lack of quality data often makes it difficult to correctly identify key enzymes through control analysis. Here, we proposed a method combining in vitro metabolic pathway analysis and proteomics measurement to find the key enzymes in threonine synthesis pathway.

**Results:**

All enzymes in the threonine synthesis pathway were purified for the reconstruction and perturbation of the in vitro pathway. Label-free proteomics technology combined with APEX (absolute protein expression measurements) data analysis method were employed to determine the absolute enzyme concentrations in the crude enzyme extract obtained from a threonine production strain during the fastest threonine production period. The flux control coefficient of each enzyme in the pathway was then calculated by measuring the flux changes after titration of the corresponding enzyme. The isoenzyme LysC catalyzing the first step in the pathway has the largest flux control coefficient, and thus its concentration change has the biggest impact on pathway flux. To verify that the key enzyme identified through in vitro pathway analysis is also the key enzyme in vivo, we overexpressed LysC in the original threonine production strain. Fermentation results showed that the threonine concentration was increased 30% and the yield was increased 20%.

**Conclusions:**

In vitro metabolic pathways simulating in vivo cells can be built based on precise measurement of enzyme concentrations through proteomics technology and used for the determination of key enzymes through metabolic control analysis. This provides a new way to find gene overexpression targets for industrial strain improvement.

**Electronic supplementary material:**

The online version of this article (doi:10.1186/s12934-015-0275-8) contains supplementary material, which is available to authorized users.

## Background

The goal of metabolic engineering is the rational design and modification of cellular metabolism for the production of useful biochemicals and bioenergy. Due to the complexity of a metabolic network and its regulation, the construction of a highly efficient cell factory often requires alteration from various aspects, such as introducing heterologous enzymes with high activity, overexpression of key enzymes, deleting genes leading to byproducts, sequence modification for the removal of end product inhibition or repression and applying evolutionary engineering strategies [1, 2]. The overexpression of key enzymes in the pathway to a desired product is often necessary for strain improvement. Several methods based on genome scale metabolic network analysis such as flux balance analysis and FVSEOF have been proposed for the identification of gene amplification targets [3–6]. However, as these methods are just based on reaction stoichiometry, all genes in a linear pathway will have equal contribution to the pathway flux and thus these methods will not be able to identify which enzymes are more important for increasing the pathway flux. A theoretical method called metabolic control analysis has been developed to quantitatively measure the impact of an enzyme on the pathway flux through flux control coefficients (FCCs) [7]. FCCs ($C{}_{i}$) is mathematically defined according to the large deviation theory [8].

$$ C{}_{i} = \frac{\varDelta J}{{\varDelta E{}_{i}}}\frac{{E_{i}^{r} }}{{J^{r} }} $$

where $J^{r}$ and $E_{i}^{r}$ represent the enzyme activity and flux of the system after enzyme concentration or activity changing, while $\varDelta J$ is the flux change and $\varDelta E{}_{i}$ is the change of enzyme activity. The large FCC value implies that the change in the enzyme concentration will lead to a big change in flux. FCCs of enzymes in a pathway can be calculated if a kinetic model is available for the pathway [9–11]. However, the quality of the kinetic models will greatly affect the metabolic control analysis results. Many kinetics models are developed based on individual enzyme kinetics which measure how metabolite concentrations affect transient enzyme rates rather than how enzyme concentrations affect the steady state pathway flux in a multi-enzyme system. Therefore metabolic control analysis from such kinetic models may not reveal the true key enzymes.

Experimental determination of FCC is possible by measuring the flux changes after perturbation on enzyme concentrations. A number of studies have dealt with the relationships between the flux of a metabolic system in vivo and the concentrations and activities of particular enzymes with the use of genetic means to alter the enzyme parameters [12, 13]. However, the in vivo studies usually confront difficulties in precise manipulation and measurement of enzyme concentrations. In contrast, alteration of enzyme concentrations in a cell free in vitro system is much easier if purified enzymes are available. The in vitro method bypasses cell walls and removes genetic regulation to enable direct access to the inner workings of the cell. Due to the unprecedented level of control and freedom of design, relative to in vivo systems, it has been widely studied for the production of biocommodities and bioelectricity in recent years [14–16]. In addition, in vitro analysis methods have also been used to guide the genetic engineering of strains for optimization of fatty acid, fatty alcohol and farnesene synthesis process [17–19].

One issue in using an in vitro system is that the enzyme concentrations must be similar with the in vivo system so that the key enzymes identified from in vitro analysis could also be effective overexpression targets. Intracellular protein concentrations could be measured through various proteomics technologies, which usually involve proteolysis of protein mixtures, followed by analysis of the peptides generated using chromatography and mass spectrometry (MS). However, due to the difference in size and amino acid composition of proteins, normal proteomics data is often suitable for comparing protein levels at different conditions but not for cross-protein comparison. To address this problem, Lu et al. [20] proposed a method called absolute protein expression measurements (APEX) to get the relative protein ratios from proteomics data. APEX relies upon correcting each protein’s mass spectrometry sampling depth (observed peptide count) by learned probabilities for identifying the peptides, like background expectation of observing each peptide in the experiment, the total sampling depth and the confidence in protein identification. And it is a robust and rapid method to quantify protein abundance without requiring construction of fusion protein libraries, labeling or internal standards. This method has been widely used for protein expression measurements [21–23].

In this research, we used label- free proteomics and APEX method to determine the absolute protein concentrations in a threonine production *E. coli* strain named *Thr*. Based on the data, we designed an in vitro multi-enzyme system to experimentally measure the FCCs of enzymes in threonine synthesis pathway. The results indicate that LysC, one of the isoenzymes of aspartate kinase, is the key enzyme for increasing threonine synthesis flux. We then overexpressed the *lysC* gene in the production strain and the threonine yield in the new strain was improved.

## Results and discussion

### Characterization of the crude enzyme extract and enzyme purification

**Figure 1:**
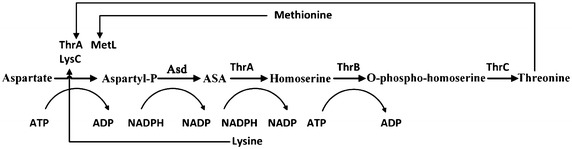
Threonine synthesis pathway from aspartate in *E. coli*. The reactions are catalyzed by aspartate kinase (ThrA, MetL, LysC), aspartate semialdehyde dehydrogenase (Asd), homoserine dehydrogenase (ThrA), homoserine kinase (ThrB) and threonine synthase (ThrC). *ASA* aspartic *β*-semialdehyde, *aspartyl*-*P* *β*-aspartyl phosphate.

**Figure 2:**
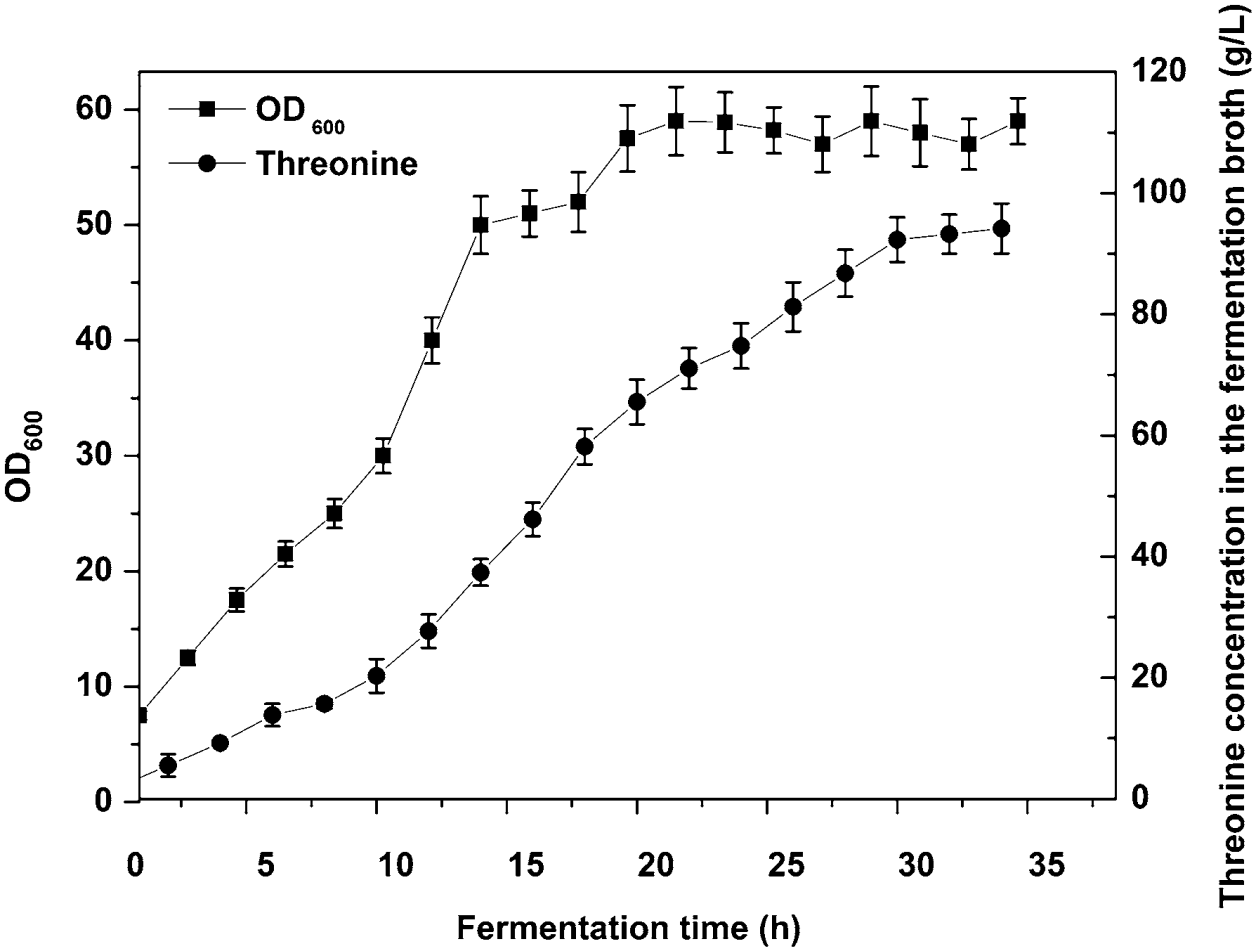
Fermentation process of threonine production stain. The cells sampled at 12 h (exponential growth phase and with high threonine production rate) were used for proteomics analysis and crude enzyme extract experiments. Two replicates of this experiment.

**Figure 3:**
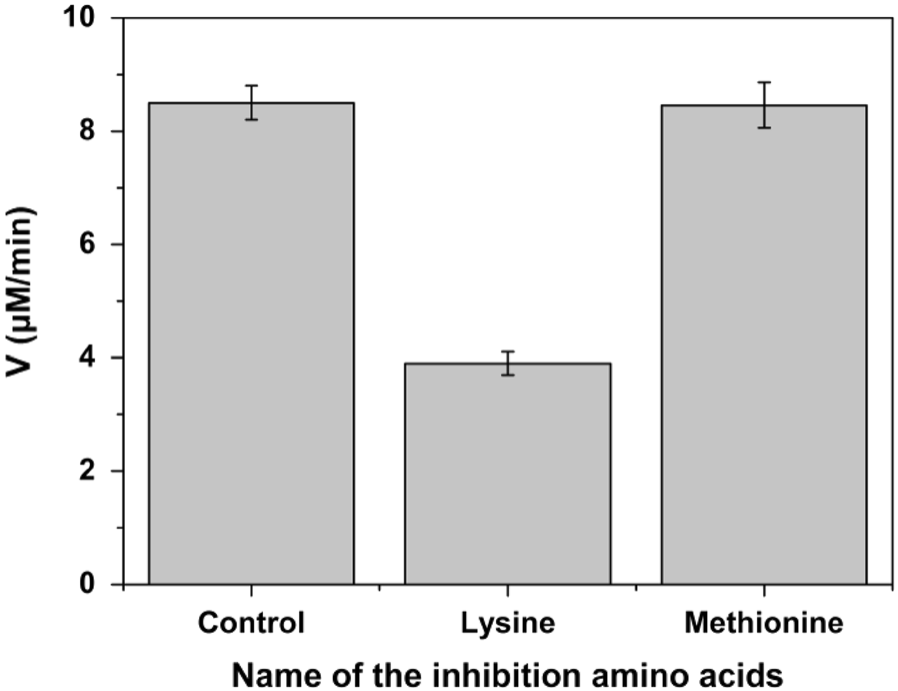
Contribution of the three isoenzymes on aspartate kinase activity in the crude enzyme extract. Control: representing the summed activity of all three isoenzymes; LysC, ThrA and MetL; Lysine: representing the activity of ThrA and MetL after the addition of Lysine to inhibit LysC; Methionine: representing the activity of ThrA and LysC after the addition of Methionine to inhibit MetL. Three replicates of this experiment.

**Figure 4:**
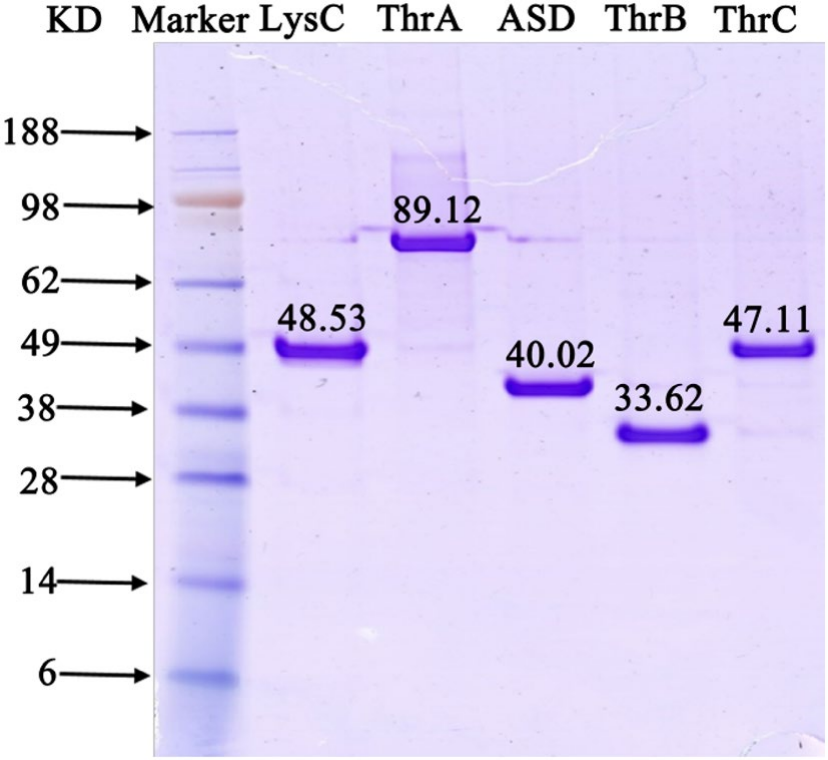
SDS-PAGE of the purified threonine synthesis enzymes.

**Fig. 5:**
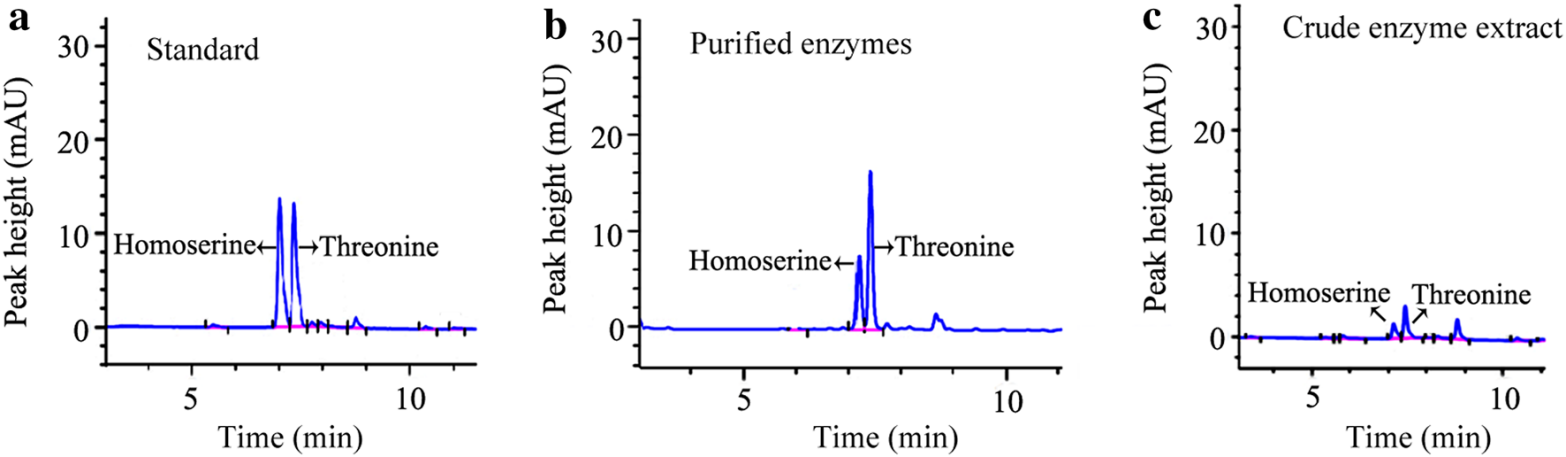
HPLC analysis of metabolites. **a** Peaks from the standard metabolites of homoserine and threonine, the *left peak* represents homoserine and the *right peak* represents threonine. **b** Homoserine and threonine produced in the in vitro reaction system by purified enzymes. **c** Homoserine and threonine produced in the in vitro reaction system constituted by crude enzyme extracts.

The five-step metabolic pathway for threonine synthesis from aspartate in *E. coli* is shown in Figure 1. The first step (aspartate kinase) was catalyzed by three isoenzymes: ThrA, LysC and MetL. In wild type strain, these three isoenzymes are inhibited by threonine, lysine and methionine respectively [20]. However, genome sequence analysis of our threonine production strain revealed a mutant in ThrA which removes threonine inhibition (unpublished data). To investigate the contribution of different isoenzymes to the first reaction, the crude enzyme extract obtained from the cells at 12 h was used. From the fermentation time course shown in Figure 2, we can see that the cells at 12 h were at the exponential growth phase and the threonine production was also at the highest rate. In vitro analysis at this period could be more helpful for identifying the true bottleneck for further improvement of threonine synthesis flux in vivo. We added 10 mM lysine and 10 mM methionine which is enough to inhibit the corresponding isoenzyme [24, 25] to the crude enzymes extract separately and checked the aspartate kinase activity change. As shown in Figure 3, there was almost no activity change when methionine was added, implying that the contribution of MetL is neglectable. When 10 mM lysine was added the reaction rate was reduced from 8.52 to 3.85 μM/min, indicating that LysC, usually responsible for lysine synthesis, made an even higher contribution toward threonine synthesis than the mutated ThrA in the production strain. Therefore, we purified LysC and ThrA as well as the other three enzymes in the threonine synthesis pathway (Asd, ThrB and ThrC). SDS-PAGE of these five enzymes was shown in Figure 4, from this figure we can see that all the purified enzymes were in the correct molecule weights and were relatively pure. We also measured the activities of the purified enzymes to make sure they were active enzymes. Figure 5a was the chromatogram of homoserine and threonine standards (the concentrations were both 1 mM), the peak at about 7.0 min was homoserine and the peak at about 7.4 min was threonine. Figure 5b showed the chromatogram of reaction with purified enzymes, two obvious peaks of homoserine and threonine could be seen which demonstrated that all of the purified enzymes were active. Figure 5c was the chromatogram of the reaction with crude enzyme extracts, and two peaks at the same time could be seen, so we could get the conclusion that all enzymes of threonine synthesis pathway in the crude enzyme extract were active.

### Determination of the absolute enzyme concentrations

**Figure 6:**
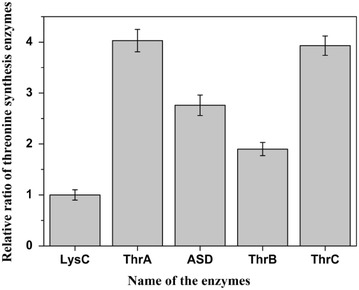
Relative ratio of the enzymes in the threonine synthesis pathway. Original proteomics data was processed by APEX method. And the relative amounts of enzymes in threonine synthesis pathway were normalized to LysC. Three replicates of this experiment.

**Figure 7:**
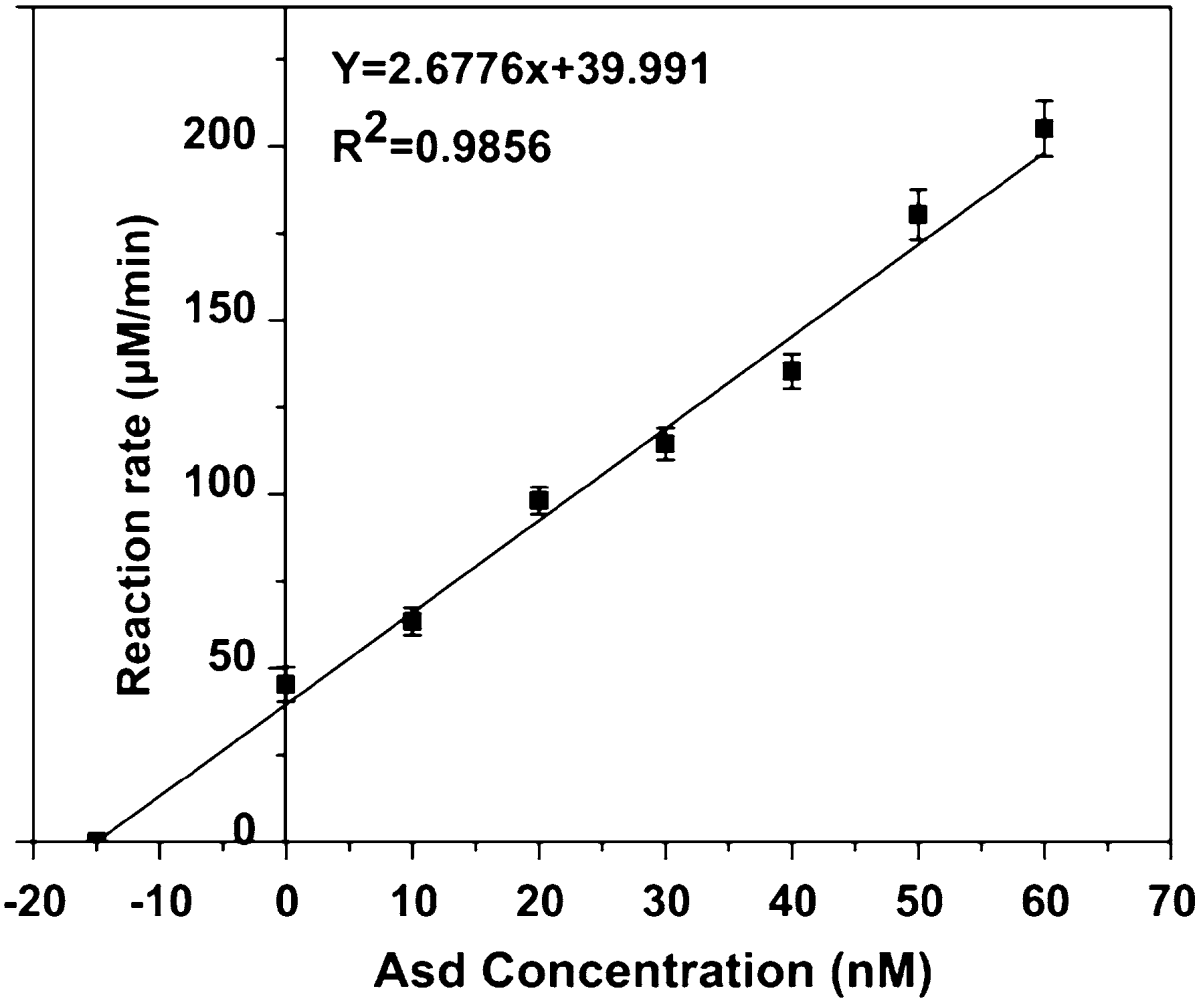
Determination of Asd absolute concentration in the crude enzyme extract. Different amounts of purified Asd were added into the diluted crude extract and the reaction rates were measured. The Asd concentration in the diluted crude extract was calculated from the intersection on X-axis and from the dilution rate the absolute Asd concentration in the crude enzyme extract was calculated. Three replicates of this experiment.

The MS data were processed using Pioplite software with protein identification and quantification. APEX method was used to process the original label-free proteomics data to get reliable data on ratios between different proteins. We chose to focus on the enzymes in the threonine synthesis pathway and the relative amount of enzymes (normalized to LysC) was shown in Figure 6. To determine the absolute enzyme concentrations, we need to measure the absolute enzyme concentration for at least one enzyme. We chose to measure Asd concentration in the crude extract as NADPH is involved in its catalyzed reaction and thus the reaction rate (proportional to the enzyme concentration) can be easily measured through fluorescent. We added different amount of purified Asd into the diluted (1:40) crude extract and measured the reaction rates. As can be seen from the result shown in Figure 7, a good linear relationship between the reaction rate and added Asd concentration was obtained. The Asd concentration in the diluted crude extract was calculated to be 15 nM from the linear relationship (the intersection on X-axis) and from the dilution rate we estimated that the absolute Asd concentration in the crude enzyme extract was 600 nM. As in the process of crude enzyme extraction, we added 10 mL enzyme assay buffer to about 0.66 mg pellet cells, the cells were diluted about 15 times. Therefore, the absolute concentration of Asd in the cells was about 9 μM. Combined with the relative enzyme ratio determined by proteomics, absolute concentrations for all enzymes in the pathway were calculated and shown in Table 1. The concentration values were in the same order of magnitude with the integrated concentration data from PaxDb database [25] which contains absolute protein concentrations from several proteomics studies on *E. coli* [20, 26–29].

**Table 1:** Measured absolute enzyme concentrations in *E. coli* cells

Enzymes	Concentrations from this study (μM)	Concentrations from PaxDb^a^ (μM)
LysC	3.24 ± 0.32	3.32
ThrA	13.09 ± 0.71	5.31
ASD	9.01 ± 0.65	12.44
ThrB	6.27 ± 0.42	5.17
ThrC	12.78 ± 0.61	7.10

^a^The concentrations from PaxDb [25] were the integrated concentration data from several proteomics studies. The protein concentrations cited here were recalculated to convert the unit from ppm to μM. The average volume of *E. coli* was set at 0.6 μm^3^ [30] and the average number of proteins in *E. coli* was set at 2.5 × 10^6^ [20].

### Key enzymes in the threonine synthesis pathway

**Figure 8:**
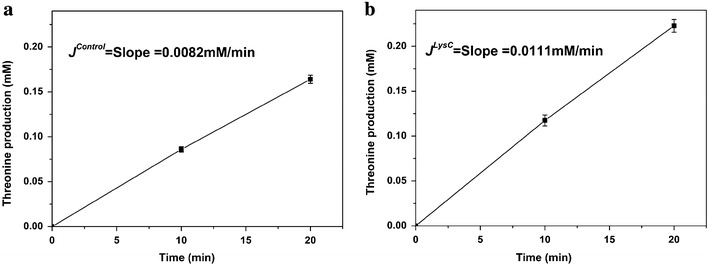
The pathway flux (*J*) in the vitro system when enzyme concentration was increased. **a** The pathway flux in the system caused by crude enzyme extract without addition of purified enzyme. **b** The pathway flux when purified LysC was added to the crude enzyme extract. The good linear relationships between threonine concentration and reaction time indicate that the in vitro pathway was at pseudo-steady state during that time period. Therefore the threonine production rates calculated from the linear relationship can represent the pathway fluxes. Three replicates of this experiment.

**Figure 9:**
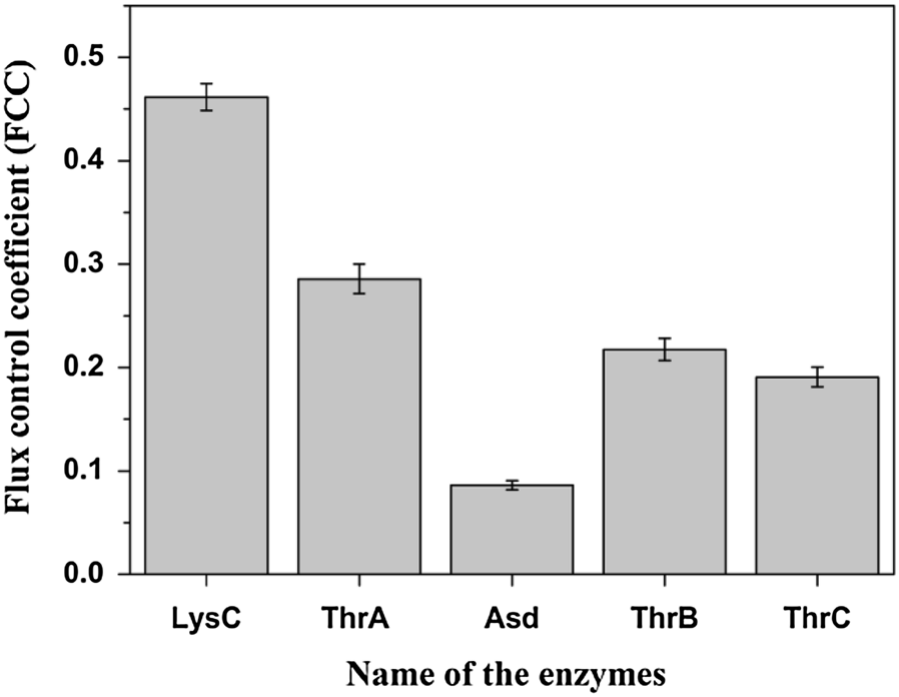
Flux control coefficients of the enzymes in the threonine synthesis pathway. LysC has the largest control coefficient and thus is the key enzyme for threonine synthesis. Three replicates of this experiment.

Key enzymes in a metabolic pathway can be determined by comparing the flux control coefficients. With the determination of the absolute enzyme concentrations in the crude extract and the availability of the purified enzymes, we were able to measure the FCCs in vitro by titration of purified enzymes into the crude extract. Based on metabolic control analysis theory, the impact of an enzyme on the pathway flux should be measured when the system reaches a steady state. However, a true steady state can only be obtained in continuously operated enzymatic reactors which are difficult to construct and operate. Frequently, a pseudo-steady state in batch reactor to calculate the flux through a pathway exists [17]. Here we also used a batch reactor to calculate the flux through the threonine pathway. The metabolites added to the reaction system include: 5 mM ASP, 5 mM ATP, 10 mM NADPH, 0.5 mM PLP. All substrate concentrations were much higher than their Km values so that the reaction rates would not be affected by the decreased substrate concentrations during the batch reaction process. To evaluate the impact of different enzymes, we added individual purified enzymes into the reaction system so that the concentration of the corresponding enzyme was increased two times. Figure 8 showed the measured threonine concentration changes over time with addition of LysC and the reaction system without addition of purified enzymes was used as control. Good linear relationships indicate the existence of pseudo-steady state during that time period and the pathway fluxes (*J*) can be calculated from the slope values of the regression line. From Figure 8b the pathway flux after addition of two times LysC was 0.0111 mM/min, while the flux of the control was only 0.0082 mM/min (Figure 8a). Other pathway flux changes were shown in Additional file (see Additional file 1: Figure S1). After the addition of purified enzymes, all pathway fluxes were increased but at different extent, indicating the different impact of enzymes. Based on the definition of FCC from large deviations theory [8], the FCCs of different enzymes were calculated from the pathway flux changes and shown in Figure 9. The flux control coefficients of LysC and ThrA were higher than the other three enzymes, implying that the first step of threonine synthesis was the control step for threonine synthesis from aspartate. This result is in agreement with the normal biochemistry knowledge that the first step of a pathway is often the rate limiting step, but is in contrast with the result from modeling analysis by Chassagnole and his colleagues [10]. They found that Asd rather than ThrA was the enzyme with the largest FCC. This may imply that some parameters (such as enzyme concentrations) used in their model might be wrong. In our research, we used the enzyme concentrations obtained from proteomics analysis of real high threonine production cells, and thus the key enzymes identified are more likely to be the true key enzymes in the cells. Interestingly, our analysis showed that the control coefficient of LysC was even larger than that of ThrA. This means that LysC is an even better gene overexpression target.

### In vivo validation of the key enzyme

**Figure 10:**
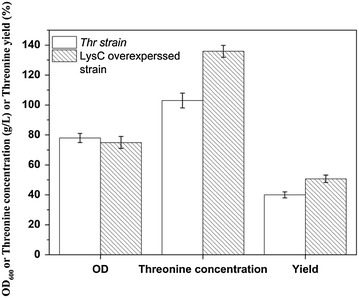
Comparison of the fermentation results of the original *Thr* strain and the LysC overexpressed strain. Two replicates of this experiment.

To demonstrate the effect of overexpression of the enzyme LysC on the threonine production we genetically engineered a new strain with a plasmid containing *lysC*. The engineered strain produced approximately 30% more threonine compared with the original strain, and the threonine yield was increased from 40 to 50% (Figure 10). This demonstrated that LysC, the key enzyme identified through in vitro metabolic control analysis, was indeed a limiting step and a successful target for increasing threonine production.

## Conclusions

In this study, a method combining proteomics measurement and in vitro metabolic pathway analysis was proposed to determine the key enzymes in threonine synthesis pathway. The precise measurement of absolute enzyme concentrations in producing cells and the possibility of accurate modification of enzyme concentrations in the in vitro pathway make it possible to quantitatively evaluate the impact of different enzymes on the pathway flux based on metabolic control analysis. Subsequently key enzymes in the pathway can be determined and the overexpression of these enzymes is more likely to be an effective strain modification strategy to improve the pathway flux in vivo. The enzyme LysC (usually responsible for lysine synthesis) showed the largest impact on threonine synthesis in our threonine production strain *Thr*. A modified strain with overexpressed LysC produced 30% more threonine compared with the original strain. It should be noted that in a different *E. coli* strain other enzymes rather than LysC might be key enzymes in threonine synthesis. However, the method developed in this study is still useful for key enzyme determination in different cells and different pathways. With the construction of even complex in vitro pathways (e.g. the whole central pathway), the method can provide versatile insights for understanding the regulation and organization of metabolic pathways and subsequently discover unusual novel metabolic engineering strategies for artificial cell factory design.

## Methods

### Materials

The substrates aspartate, NADPH, ATP, PLP and the inhibitors for the enzymes reaction lysine, methionine, and threonine were purchased from Sigma (USA), the restriction enzymes and PCR mix were purchased from Fermentas (USA). Na_2_HPO_4_ and other organic solvents used for HPLC were purchased from Merck (German). Other chemicals used in this article otherwise demonstrated were purchased from Solarbio (Beijing, China). The kits and markers used for the construction of clones were from Transgen (Beijing, China). The protein markers and SDS-PAGE gels were purchased from life technologies (USA).

### The fermentation of threonine production strains and sampling

The crude enzyme extract was prepared from threonine production strain *Thr*. The cells were grown 8 h in LB shake flask cultures and then were inoculated in 3.5 L fermentation medium (composed of 80 g glucose, 4 g (NH_4_)_2_SO_4_, 0.02 g Ile, 2 g KH_2_PO_3_, 1 g MgSO_4_·7H_2_O, 4 g yeast extract powder, 0.015 g FeSO_4_·7H_2_O, 0.015 g MnSO_4_·H_2_O and 1 g lycine of every 1 L medium). The fermentation temperature was 37°C, pH was controlled at 7.0 and glucose concentration in the fermentation was controlled between 0 and 5 g/L. The fermentation broth was sampled every 2 h for threonine production and cell density analysis. A sample obtained at exponential growth phase and with high threonine production rate was used for proteomics analysis and crude enzyme extract experiments. The cells were harvested by centrifugation at 6,000*g* for 30 min at 4°C. For the crude enzyme extract preparation, the cells were washed three times with enzyme assay buffer (23 mM Tes, 114 mM KCl, 6 mM MgCl_2_, pH 7.5) and were disrupted by high pressure homogenizer. The cells lysate was centrifuged at 12,000*g* for 30 min at 4°C to remove cell debris. Then the supernatant was dialyzed to remove small molecules. The final crude enzyme extracts were stored at −80°C after flash freezing with liquid nitrogen for further proteomics analysis and crude enzyme extract experiments.

### Proteomics analysis

The cells were the same as those for preparation of the cell crude extract. About 100 mg wet mass were processed through single tube whole cell lysis and protein digestion. Cells were frozen by Liquid nitrogen immediately after centrifugation and washed three times with phosphate buffer (pH 7.0). The cells were then resuspended in a lysis buffer (Tris–HCl 0.1 M, pH 7.6, DTT 0.1 M) and broken with sonication cracking on ice with the parameters set as follows: 5 s on, 5 s off, total 15 min using the sonification device made in Nanjing. After sonication the supernatant was centrifuged for 20 min at 16,000*g*. The supernatant was filtered through sterile membrane filter (0.22 μm) and then transferred into a new tube. After boiling for 10 min the supernatant was stored at −80°C prior to sample cleanup if not for immediate use. The protein pellet extracted from previous step was quantitated using 2D-Quant Kit (purchased from GE healthcare). And then protein pellet from previous step was washed by UA buffer [8 M Urea dissolved in 0.1 M Tris–HCl (pH 8.5)] and dissolved in digestion buffer [100 mM TEAB (triethylammonium bicarbonate)] to a final concentration of 1 mg/mL. Equal aliquots were then digested with trypsin overnight at 37°C (Promega).

A NanoLC system (NanoLC 2D Ultra, Eksigent) equipped with Triple TOF 5600 mass spectrometer (AB SCIEX, USA) was used for analysis. Peptides were trapped on NanoLC pre-column (Chromxp C18, 3 μm, size 0.35 × 10 mm) and then eluted onto an analytical column (chromxp C18, 3 μm, size 0.075 × 150 mm) and separated by a 60 min gradient from 5 to 60% Buffer B (98% ACN, 2% H_2_O, 0.1% FA) at a flow rate of 300 nL/min. Full-scan MS was performed in positive ion mode with nano-ion spray voltage of 2.5 kv from 350 to 1,500 (m/z). For IDA, survey scans were acquired in 250 ms and as many as 30 product ion scans were collected if exceeding a threshold of 125 counts per second (counts/s) and with a + 2 to a + 5 charge-state.

### Plasmid construction

The genomic DNA from *Thr* was used as the template for amplification of the threonine synthesis genes. The pET28a based plasmids were constructed for the purification of the enzymes with six histidine residues at N-terminus. The genes were amplified using primers shown in Table 2, after amplification the genes were inserted into pET28a vector using restriction enzymes and T4 DNA ligase. The plasmids and strains used in this study were shown in Table 3.

**Table 2:** PCR primers used in this study

Enzyme name	Prime name	Primer sequence (5′–3′)
ThrA	*thrA*_F1	GGGAATTCCATATG ATGCGAGTGTTGAAGTTCGGCGGTA
	*thrA*_R1	CCGGAATTCTCAGACTCCTAACTTCCATGAGAGG
LysC	*lysC*_F1	GGGAATTCCATATG ATGTCTGAAATTGTTGTCTCCAAAT
	*lysC_*R1	CCGGAATTCTTACTCAAACAAATTACTATGCAGT
ASD	*asd*_F1	CTAGCTAGCATGAAAAATGTTG GTT TTATCGGCT
	*asd*_R1	CCGGAATTCTTACGCCAGTTGACGAAGCATCCGA
ThrB	*thr*B_F1	GGGAATTCCATATGATGGTTAAAGTTTATGCCCCGGCTT
	*thr*B_R1	CCG GAATTCTTAGTTTTCCAGTACTCGTGCGCCC
ThrC	*thr*C_F1	GGGAATTCCATATGATGAAACTCTACAATCTGAAAGATC
	*thr*C_R1	CGCGGATCCTTACTGATGATTCATCATCAATTTA
LysC	*lysC*_F2	CCG GAATTCATG ATGTCTGAAATTGTTGTCTCCAAAT
	*lysC_*R2	CGC GGATCCTTACTCAAACAAAT TACTATGC AGT

**Table 3:** Strains and plasmids used in this research

Strains and plasmids	Description
*Thr*	The genome of this strain was used as the template for amplification of threonine synthesis genes
*E. coli* DH5a	This strain was used for the clone of the plasmid
*E. coli* BL21	This strain was used for enzyme protein expression
pET28a-*thrA*	N-terminal His-tagged *thrA*, inserted between NdeI and EcoRI sites of pET 28a (+)
pET28a-*lysC*	N-terminal His-tagged *lysC*, inserted between NdeI and EcoRI sites of pET 28a (+)
pET28a-*asd*	N-terminal His-tagged *asd*, inserted between NheI and EcoRI sites of pET 28a (+)
pET28a-*thrB*	N-terminal His-tagged *thrB*, inserted between NdeI and EcoRI sites of pET 28a (+)
pET28a-*thrC*	N-terminal His-tagged *thrC*, inserted between NdeI and BamHI sites of pET 28a (+)
pWSK29-*lysC*	*lysC*, inserted between EcoRI and BamHI sites of pWSK29

### Enzyme expression and purification

To purify the enzymes, the corresponding plasmids were introduced into *E. coli* BL21 (DE3). Single colonies were grown in LB medium with 50 μg/mL Kanamycin at 37°C until OD_600_ of the culture reached 0.6–0.8. Cultures were cooled to 16°C, and IPTG was added to a final concentration of 1 mM. After further growth at 16°C for 14–16 h, the cells were harvested by centrifugation at 6,000*g* for 30 min at 4°C, the supernatant was discarded. The pellet was suspended with buffer A (150 mM Tris–HCl, 150 mM NaCl and 20 mM imidazole, 1 mM DTT, pH 7.5) and the cells were disrupted by high pressure homogenizer. Then the cell lysate was centrifuged at 12,000*g* for 30 min at 4°C to remove cell debris. The supernatant was loaded onto Ni–NTA His-Bind column (GE Healthcare). The purification process was conducted on the protein purification machine AKTA purifier 10 with linear gradient imidazole from 20 mM to 500 mM in buffer. The fraction with higher OD_280_ was selected for SDS-PAGE. The fraction with pure protein was selected for dialysis in order to remove imidazole. After purification, the purified enzymes were quantitated using 2D-Quant Kit (purchased from GE healthcare) and purity was accessed by SDS-PAGE. The freshly purified proteins were stored at −80°C until use.

### Assay of the enzyme activities

The activities of aspartate kinase in the crude enzyme extract were measured in a coupled assay with pyruvate kinase (PK) and lactate dehydrogenase (LDH) as described by Chassagnole and his colleagues [24] by following the NADH oxidation at 340 nm at 37°C. The reaction mixture contained in 200 μL of assay buffer, 5 mM aspartate, 5 mM ATP, 1.5 mM phosphoenolpyruvate, 0.3 mM NADH, 2.5 U PK, 2.5 U LDH and appropriate amount of crude enzyme extract. To investigate the contribution of different isoenzymes to the first reaction, 10 mM lysine and 10 mM methionine (enough to inhibit the corresponding isoenzyme) were added to the reaction system separately.

The activities of purified enzymes were assayed with detection of threonine after all five enzymes added. The reaction systems were as follows: 5 mM ASP, 5 mM ATP, 10 mM NADPH, 0.5 mM PLP, the concentrations of LysC, ThrA, Asd, ThrB, ThrC were all 100 nM. The reaction was conducted at 37°C for 0.5 h. The reaction liquid was heated at 100°C for 10 min to inactivate the enzymes when the reaction finished. After inactivation the liquid was centrifuged at 12,000*g* for 20 min and used for the assay of threonine by HPLC using the method provided by Agilent Company. The mobile phase A was 40 mM Na_2_HPO_4_, pH 7.8, mobile B was ACN:MeOH:water (45:45:10, v/v/v). The flow rate was 2 mL/min. The same procedure was used for crude enzyme extract activity analysis. There were three replicates of each assay.

### In vivo validation of the key enzyme

For the in vivo validation of the key enzyme identified from the in vitro enzyme reactions, the overexpression plasmid was constructed using pWSK29 and primers named as *lysC*_F2 and *lysC*_R2 (shown in Table 2) in order to distinguish them from those used for construction of LysC expression plasmid. *Thr* strain containing the LysC overexpression plasmid was fermented and the fermentation process was the same as those in the fermentation of *Thr* strain for samples of crude enzyme extracts preparation.

## Additional files

Additional file 1:
**Figure S1.** The pathway flux (*J*) in the in vitro system when one enzyme concentration was increased. (**A**) The pathway flux when purified ThrA was added to the crude enzyme extract. (**B**) The pathway flux when purified Asd was added to the crude enzyme extract. (**C**) The pathway flux when purified ThrB was added to the crude enzyme extract. (**D**) The pathway flux when purified ThrC was added to the crude enzyme extract.
