# Supplementary material for: Determination of key enzymes for threonine synthesis through in vitro metabolic pathway analysis
Source: Microb Cell Fact. 2015 Jun 13;14:86. doi: 10.1186/s12934-015-0275-8 (PMC4465468; doi:10.1186/s12934-015-0275-8)
Supplement: Additional file 1: — Figure S1. The pathway flux (J) in the in vitro system when one enzyme concentration was increased. (A) The pathway flux when purified ThrA was added to the crude enzyme extract. (B) The pathway flux when purified Asd was added to the crude enzyme extract. (C) The pathway flux when purified ThrB was added to the crude enzyme extract. (D) The pathway flux when purified ThrC was added to the crude enzyme extract. [file 12934_2015_275_MOESM1_ESM.docx]

## Supplementary to the manuscript

**Figure S1 The pathway flux (*J*) in the *in vitro* system when one enzyme concentration was increased. (A**) The pathway flux when purified ThrA was added to the crude enzyme extract. (**B**) The pathway flux when purified Asd was added to the crude enzyme extract. (**C**) The pathway flux when purified ThrB was added to the crude enzyme extract. (**D**) The pathway flux when purified ThrC was added to the crude enzyme extract.

**
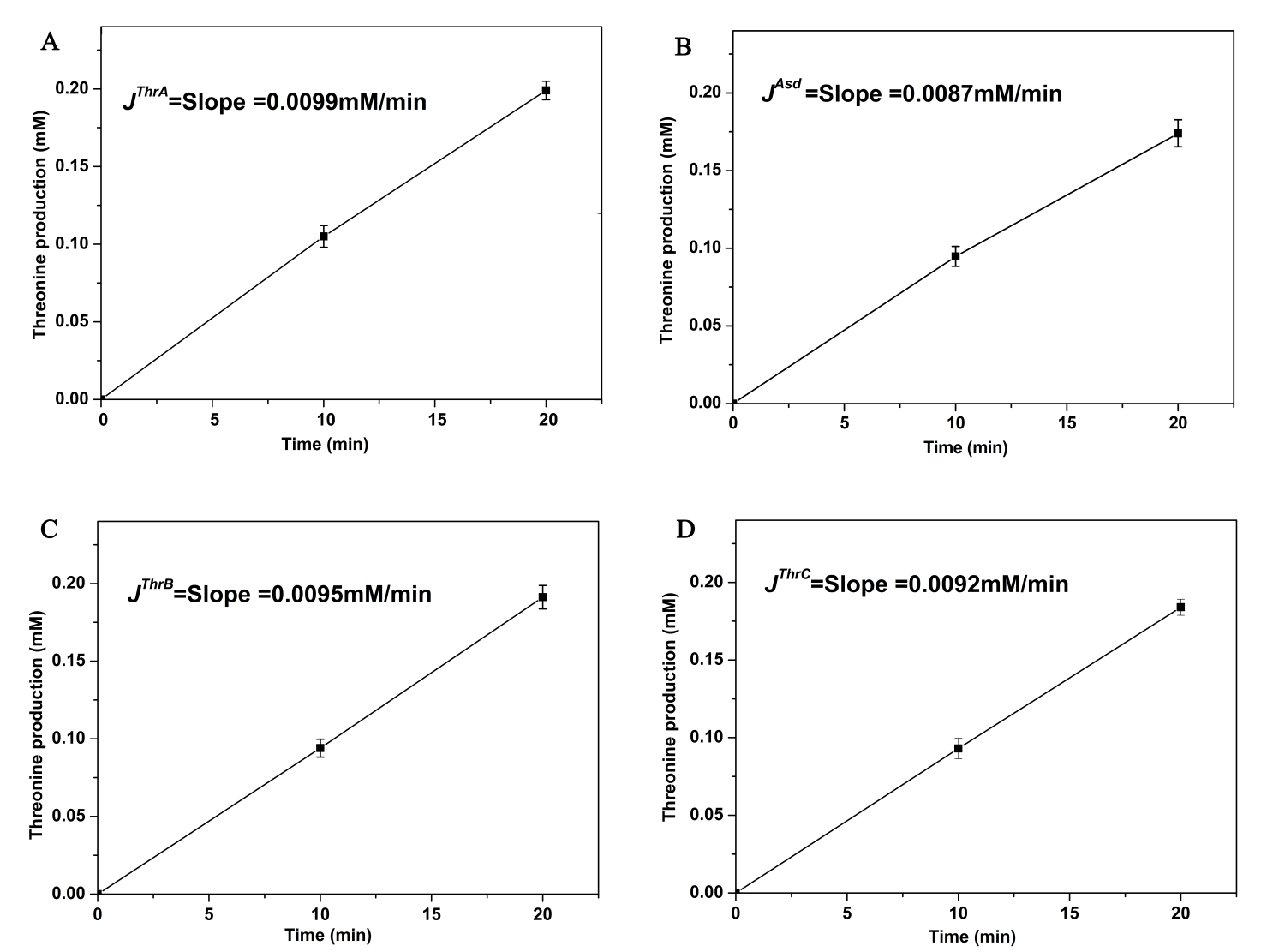
**
